# Wavefront Changes during a Sustained Reading Task in Presbyopic Eyes

**DOI:** 10.3390/s24123866

**Published:** 2024-06-14

**Authors:** Ebrahim Safarian Baloujeh, José M. González-Méijome

**Affiliations:** 1 Consejo Superior de Investigaciones Científicas (CSIC), Instituto de Nanociencia y Materiales de Aragón (INMA), 50009 Zaragoza, Spain; 2 Departamento de Física Aplicada, Universidad de Zaragoza, 50009 Zaragoza, Spain; 3 Clinical and Experimental Optometry Research Laboratory (CEORLab), Department and Center of Physics—Optometry and Vision Science, School of Science, 4710-057 Braga, Portugal; jgmeijome@fisica.uminho.pt

**Keywords:** ocular wavefront, Zernike coefficients, aberrometry, HOA

## Abstract

The objective of this study was to assess the effect of sustained reading on the temporal changes in the wavefront error in the presbyopic eye. The wavefront aberration of the eyes was measured using an IRX3 Shack–Hartmann aberrometer before and after (immediately, 5 min, and 10 min after) a reading task. Temporal changes in $C_{2}^{0}$, $C_{4}^{0}$, and $C_{3}^{-1}$ coefficient values of the eyes were plotted, showing a predominant number of V-shaped patterns (for $C_{4}^{0}$ and $C_{3}^{-1}$) and inverse V-shaped patterns (for $C_{2}^{0}$) among the study group, and the percentages (between 27 and 73%) were reported. The median of the total RMS of aberrations and the RMS of HOA (higher-order aberrations), which included comatic (3rd order) and spherical-like aberrations (4th and 6th order), increased immediately after finishing the near-vision reading task and then decreased. The median of RMS of comatic aberrations had a similar pattern of variations, while the median of RMS of spherical-like aberrations displayed an opposite pattern. Simulating the aberration changes due to lens decentration caused by relaxed zonules during 4 D accommodation in an eye model demonstrated that the expected range of changes for the vertical coma and spherical aberrations are in the order of 0.001 and 0.01 μm, respectively, which could justify why the observed changes were not statistically significant. The observed dynamic changes in HOA might be linked to the biomechanical characteristics and alterations in the displacement of the crystalline lens following prolonged near-vision tasks in presbyopic people. Although some predominant patterns under some conditions were shown, they exhibit considerable inter-subject and inter-ocular variability. This might be due to slight misalignments while fixating on the internal extended object in the aberrometer.

## 1. Introduction

In today’s world, humans need to perform durable tasks that require accommodation, especially now that computers and digital devices have found an essential role in everyday life. The term sustained near vision refers to the ability of the eye to maintain objects at a close distance in clear focus for an extended period of time. The accommodative system undergoes physiological changes during and after sustained tasks. For nearly 350 years, different theories have been proposed to explain the accommodation mechanism [1] but the most widely accepted theory was presented by Helmholtz [2]. According to him, the accommodation process involves a contraction of the ciliary muscle, which decreases zonular tension; subsequently, the lens’s anterior curvature gets steeper, the axial thickness increases, and its diameter decreases. During accommodation, when zonular fibers are relaxed, the crystalline lens could decenter down under gravity force, eventually creating changes in visual quality [3]. For the first time, Hess documented this phenomenon under intense accommodation, which was observable entoptically. The observed displacement, reaching a magnitude of 0.3 mm, demonstrated the impact of the gravitational force acting on the eye lens that is inadequately supported by the relaxed zonula. Later, ultrasonography research conducted by Coleman [4] supported Hess’s findings. Glasser and Kaufman [5] also validated the translational movement of the lens as observed by Hess in monkeys during centrally stimulated accommodation.

By aging, the eye loses its accommodating ability, gradually translating into presbyopia. Hess (and later Gullstrand) tried to describe the mechanism of presbyopia. It has been hypothesized in the theory of Hess–Gullstrand [6,7] that the ciliary muscle may retain a significant amount of its contractile ability as we age but is unable to reshape the sclerosed crystalline lens structure. Strenk et al. [8] using MRI demonstrated that the human ciliary muscle retains its ability for movement throughout an individual’s lifespan.

On the other hand, the nucleus of a crystalline lens is stiffer than its cortex in presbyopes [9]. Considering that the cortex of the lens keeps some elasticity and ciliary muscle can yet contract in a presbyopic eye, the lens may slide around the nucleus changing its equatorial diameter [10]. Therefore, it is reasonable to anticipate slight optical alterations in presbyopic eyes when they are subjected to stimuli that require sustained accommodation.

Nearwork-induced transient myopia (NITM) is a long-studied optical effect that occurs when the eyes are involved in a close-range activity for an extended duration and hardly relax the accommodation when no longer focused on a near object. The observed amplitude of NITM varied from 0.2 to 0.6 D and may be triggered by near-work activities at a distance of 0.12 to 0.25 m, lasting between 3 and 60 min [11]. The first investigation in this field was carried out by Lancaster and Williams [12]. The assessment of the far point was conducted before and after the completion of a prolonged near-vision task. The resulting myopia was subjectively measured at both distant and close-viewing distances in visually normal volunteers of various ages. In their experiment, the return of the far point to the baseline value required a duration of up to 15 min. A spasm of accommodation, which they called retarded relaxation, is thought to be one of the reasons for NITM. The duration of nearwork and refractive groups are also affecting NITM. In contrast to individuals with normal vision, those with early-onset myopia and late-onset myopia exhibit increased vulnerability to NITM, resulting in less accommodative precision for distant vision after prolonged close visual tasks [13]. Ciuffreda and Lee [14] extended their previous experiment in order to include an extended duration of near visual tasks in a lighting condition that closely resembles real-life settings. They hypothesized that NITM caused by accommodative hysteresis over an extended period of time has a role in myopigenesis.

Besides NITM, a small deformation of the lens cortex in presbyopes, together with a downward translation of the lens during accommodation, may contribute to changes in higher-order aberrations (HOA) as well. These changes can be studied using the wavefront aberrometry technique during sustained near visual tasks such as reading a book. Gomes and Franco [15] showed that engaging in sustained nearwork has the potential to induce alterations in the internal optics of the eye.

Reviewing the literature, there are studies that have investigated accommodation-related changes in the eye. Ninomiya et al. [16] reported no significant changes in the root mean square (RMS) values of the total higher-order aberrations with accommodation for 4-mm and 6-mm pupils; however, Li et al. [17] reported that with 5.0 mm pupils, the RMS of total HOA changed with accommodation. Meanwhile, most studies confirmed that primary spherical aberration (4th order) decreases with the increase in accommodation. Glasser and Campbell [18] reported a similar result for the change in spherical aberration with age in vitro using a lens stretcher. López-Gil and Fernández-Sánchez [19], based on theoretical and ray-tracing calculations performed on an accommodating eye model, predicted that secondary spherical aberration (6th order) rises with accommodation. Other researchers also confirmed this in their reports [16,19,20]. Vasudevan et al. [21] demonstrated that spherical aberration is higher in myopic subjects than emmetropic subjects after performing sustained reading. In contrast to spherical aberrations, comatic aberrations (3rd order) exhibit complicated patterns with accommodation that differ among people [20,22,23,24]. The impact of HOAs on the overall aberrations in the eye is comparatively lower but may lead to glare, blurring, night-vision problems, halos, and diplopia [25].

Regarding the effect of luminance during sustained work, Johnson [26] found that as brightness decreases, the accommodation range decreases and eventually reaches a “fixed focus” that corresponds to an intermediate distance under low luminance conditions. According to the findings of Lara et al. [27], a decrease in the amplitude of accommodation during low-light conditions, known as night presbyopia, is not solely attributed to a reduction in depth of focus resulting from pupil dilation. Rather, they found that this phenomenon is significantly influenced by the decrease in retinal illumination. Therefore, they conclude that reducing lighting conditions will worsen the decrease in amplitude of accommodation associated with aging. Wolska and Śwituła [28] showed that an increase in surrounding luminance reduced the amplitude of accommodation in VDT (Video Display Terminal) operators. Using the adjustable LED (Light Emitting Diode) task lights reduced user complaints of eye discomfort and fatigue [29]. Benedetto et al. [30] studied the effect of luminance and ambient illuminance on visual fatigue and alertness. They found that when subjects read on a computer with high screen brightness, the number of blinks decreased compared to reading on low screen brightness. It was concluded that the higher the level of screen luminance, the higher the visual fatigue. Alertness was also increased with increasing levels of screen luminance or ambient illuminance increase. Based on the results of Orduna et al. [31], it was also observed that after a five-minute reading activity, the higher-order aberrations exhibited variations from their initial state across all lighting situations and reading devices.

Although the impact of accommodation on ocular aberrations has been studied, very little research has been conducted to investigate the effects of sustained near-vision tasks on wavefront aberrations. The purpose of this study was to evaluate the impact of sustained reading on temporal changes in the wavefront error in presbyopic subjects after a sustained reading activity in high- and low-lighting conditions.

## 2. Materials and Methods

When it comes to assessing the optical quality of the eye, the Hartmann–Shack method is the one that is used more often than any other wavefront-sensing technique [32]. In this method, a laser beam is projected on the fovea and the vergence of the light reflected back, after passing through the eye suffering aberrations, is analyzed. Using an array of microlenslets, the beam is split into several beams and an array of spots on the CCD sensor is formed. The wavefront’s shape is calculated by comparing the locations of each point to their non-distorted counterparts [33]. In the present study, we utilized the IRX3 commercial Hartmann–Shack aberrometer (Imagine Eyes, Orsay, France). This device measures aberrations at a wavelength of 780 nm using a 1024 lenslet array. The refractive errors that this aberrometer can measure range from −15 D to +20 D of the sphere and −10 D to +10 D of the cylinder. For the desired pupil size, the software fits the measured wavefront to a series of Zernike polynomials with different weights given by the Zernike coefficients up to the 10th order for larger pupils (IRX3 datasheet, Imagine Eyes). The values of those coefficients were used to compute the effect of the near-vision task on presbyopic eyes.

A luminance meter (Konica-Minolta, LS-110, Tokyo, Japan) was used to measure the luminance of the screen where the reading task was presented and an illuminance meter (Konica-Minolta, T-10) was used to measure the illuminance in the ocular plane at the task distance.

### 2.1. Subjects and Experimental Conditions

Thirteen healthy volunteers with ages ranging from 45 to 57 years (mean 49 ± 4) were recruited for this study. The subjects were instructed to abstain from caffeine and electronic devices for a minimum of one hour prior to the measurements. They were asked to perform a near-vision reading task under two lighting conditions: first, ambient light was maximum (255.81 lux, high lighting condition), and the brightness of the reading device was set to the highest value (where pointing a luminance meter to a white circle and a black circle yielded 362.54 cd/m^2^ and 2.23 cd/m^2^, respectively, and the Michelson contrast equaled 0.987). Second, the ambient light was dimmed (0.97 lux, low lighting condition), and the brightness of the device was set to the lowest value (the luminance of a white circle and a black circle were 5.46 cd/m^2^ and 0.02 cd/m^2^, respectively, and the Michelson contrast equaled 0.992).

A laptop (Microsoft Surface Book Pro 2, Microsoft Corporation, Redmond, WC, USA) with a screen size of 3000 × 2000 pixels was used for performing this reading task. The screen area was limited by a mask to a reading area of 21 cm wide × 14 cm high to control the light coming from the margins of the text. The reading text was written in black characters on a white background in Microsoft Word 2019 on a landscape sheet with Arial 11 font, line spacing 1.5, and a margin of 1.27 cm on all sides, while the zoom level was set at 100%.

### 2.2. Measurements

**Figure 1:**
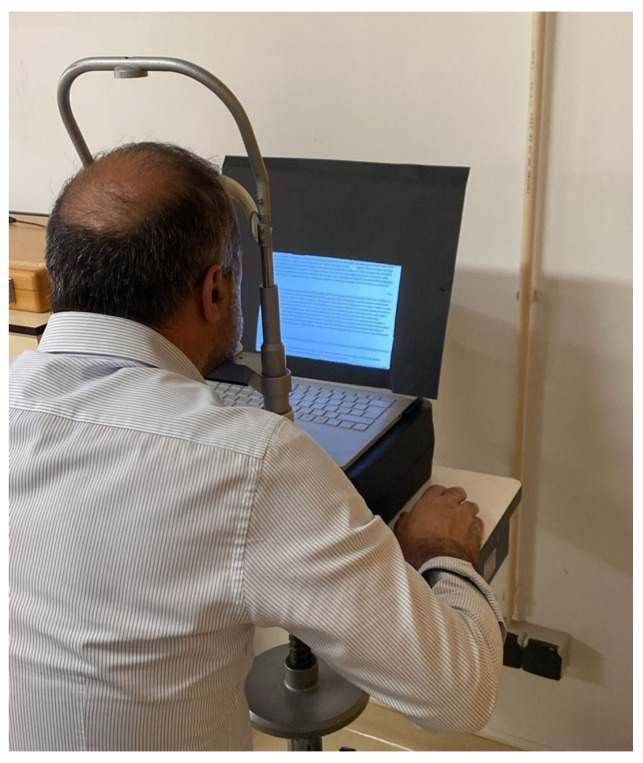
A presbyope undergoing sustained near-vision task.

Before beginning the reading, baseline aberrometry was consistently performed by the same observer upon the participant’s arrival. Baseline aberrometry was always performed by the same observer upon the arrival of the participant before starting the reading. Participants rested their chin and forehead on a head-movement stability tool at a distance ranging from 18 cm to 45 cm away from the reading device. This distance was chosen in a way that made the subject’s eye accommodate at their maximum residual capacity. To reach this point, the distance was adjusted until the nearest point where the text was sharp and started to become blurred. Any relaxation in the accommodative system under these conditions would stop the reading task. The subjects were asked to read a text for 20 min in the above-mentioned high- and low-lighting conditions. An appealing text on the history of humanity from Yuval Noah Harari (Sapiens) was chosen to keep the observers engaged with the task. As they read, they scrolled down the text using a handheld mouse. The subjects confirmed they were engaged and interested in the storytelling. Figure 1 demonstrates the sustained near-vision task condition.

Aberrometry measurements were performed before the reading activity, right after doing the activity, and 5 min and 10 min after finishing the activity. Measurements were made in triplicate (12 per eye) at each time point and then averaged for subsequent analysis. The order of measurement of the left and right eyes was random. To prevent an increase in tear film aberration that may potentially arise during a prolonged interval between blinks, the subjects were told to blink before taking measurements [34]. Aberrometric data for each eye were rescaled to the minimum round pupil value found among the series of 12 measurements per eye. The fixation target used an extended object (E-letter) in black color on a white background, with the size equivalent to 0.5 decimal acuity, i.e., 0.3 logMAR, or 10 arcmin from top to bottom.

**Figure 2:**
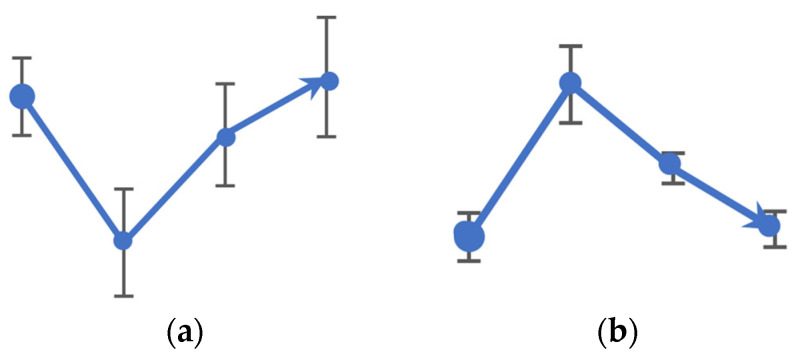
Different patterns of behavior of the coefficients: (**a**) the V-shaped pattern; (**b**) the inverse V-shaped pattern.

Figure 2 demonstrates the different behavior patterns of the coefficients during post-task measurements. Our interested patterns are those similar to the V-shaped and the inverse V-shaped patterns among flat or randomly changed patterns. While the flat and random patterns are associated with no changes or random changes, the V-shaped pattern (Figure 2a) depicts a trend toward negative values of the Zernike coefficient after performing the task, which recovers back afterward, with the opposite occurring for the inverse V-shaped pattern (Figure 2b).

### 2.3. Statistical Analysis

SPSS 25 (SPSS Inc., IBM Corporation, Somers, NY, USA) was used to analyze the data, and Excel 2019 ((Microsoft Corporation, Redmond, WA, USA) was utilized to demonstrate the changes. Since some of the data were not distributed normally, the medians of the RMS data were plotted, and Friedman’s test was used to find statistically significant changes, before and after the reading task in different lighting conditions. Linear regression was also used to report the trends of changes in the Zernike coefficients.

## 3. Results

**Figure 3:**
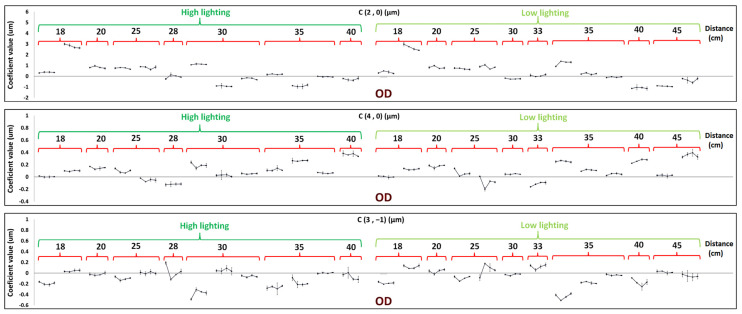
$C_{2}^{0}$, $C_{4}^{0}$, and $C_{3}^{-1}$ coefficient values of the right eyes of subjects, before, right after, after 5 min, and after 10 min of near-vision task, respectively, for high- and low-lighting conditions, with respect to distance to the stimulus. Each pattern is for one subject.

**Figure 4:**
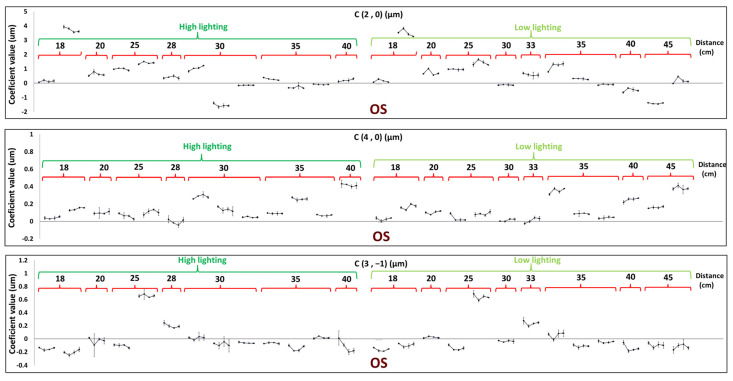
$C_{2}^{0}$, $C_{4}^{0}$, and $C_{3}^{-1}$ coefficient values of the left eyes of subjects, before, right after, after 5 min, and after 10 min of near-vision task, respectively, for high- and low-lighting conditions, with respect to distance to the stimulus. Each pattern is for one subject.

Figure 3 and Figure 4 plotted the averaged Zernike coefficients $C_{2}^{0}$, $C_{4}^{0}$, and $C_{3}^{-1}$ from the three repeated measures for right and left eyes, rescaled for a common pupil size. There was a slight inverse V-shaped trend for defocus aberrations ($C_{2}^{0}$ coefficient) in the high lighting condition that increased right after performing visual tasks and recovered to the baseline value in the subsequent measurements in 42% of right and left eyes, while 58% of them did not show such a trend and had either flat or random change patterns. The percentage for the low lighting condition was 54%, against 46% of eyes showing flat or random fluctuations.

On the other side, findings are different for the 4th order spherical coefficient ($C_{4}^{0}$) and for the vertical comatic coefficient ($C_{3}^{-1}$), showing sharper V-shaped trends right after the sustained near-vision task and inflexion thereafter for 10 min of recovery time. The $C_{4}^{0}$ coefficient undergoes a decrease right after the task and then increases in 46% of the left and right eyes in the high lighting condition, while 54% of them do not show this trend and have either flat or random change patterns. The same V-shaped trend was only observed for $C_{4}^{0}$ in 27% of left and right eyes in the low lighting condition, and in 73% of them, this trend was not seen. For vertical coma ($C_{3}^{-1}$), we see a V-shaped pattern in 42% of right and left eyes in the high lighting condition, while in 58% of them, this pattern was not seen, and the same decrease and then increase trend was seen in 73% of right and left eyes under the low lighting condition, while in 27% of them, this pattern was not the case. Table 1 summarizes the frequencies of patterns encountered under the different conditions.

To quantitatively compare these changes, ΔZ values are calculated by subtracting two consecutive values of a Zernike coefficient; for instance, the coefficient value measured right after performing the reading task is subtracted from that coefficient’s value before the reading task and called ΔZ_1_. Hence, ΔZ_1_ represents the Zernike coefficients change due to accommodation, while ΔZ_2_ and ΔZ_3_ represent relaxation/recovery of accommodation after 5 and 10 min of finishing the reading task, respectively.

**Figure 5:**
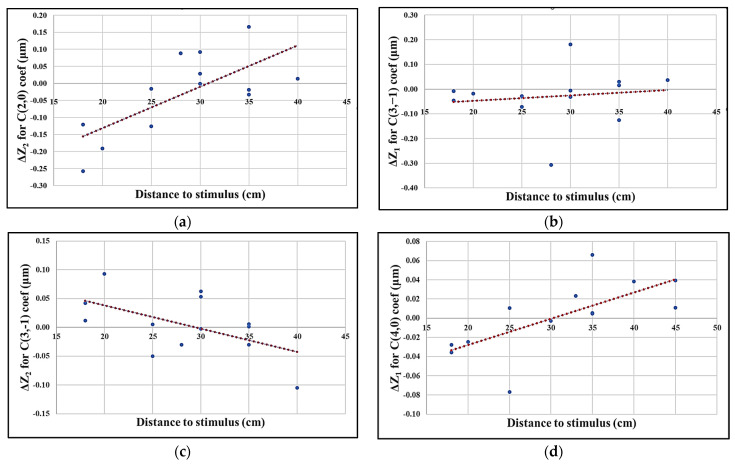
Changes in the Zernike coefficients (ΔZ values as represented in Figure 2) with distance to stimulus: (**a**) ΔZ_2_ in defocus in high lighting condition in the left eyes; (**b**) ΔZ_1_ in vertical coma in high lighting condition in the right eyes; (**c**) ΔZ_2_ in vertical coma in high lighting condition in the left eyes; (**d**) ΔZ_1_ in spherical aberration in low lighting condition in the left eyes.

Analyzing the change in ΔZ values belonging to the Zernike coefficients with distances at which the reading tasks were performed yielded graphs such as Figure 5. The change in ΔZ_2_ of the defocus coefficient $C_{2}^{0}$ in the high lighting condition shows that, as expected, the shorter the distance to the laptop, the larger the change observed immediately after the task (relaxation) (R^2^ = 0.51). Contrary to what was expected, the vertical coma does not change much with distance or maybe the changes expected are indeed of this order of magnitude (R^2^ = 0.02); however, the ΔZ_2_ change in $C_{3}^{-1}$ in the high lighting with distance is quite remarkable (R^2^ = 0.30). Regarding spherical aberration coefficients, $C_{4}^{0}$ changes occur more in the low lighting condition with distance (R^2^ = 0.48).

**Figure 6:**
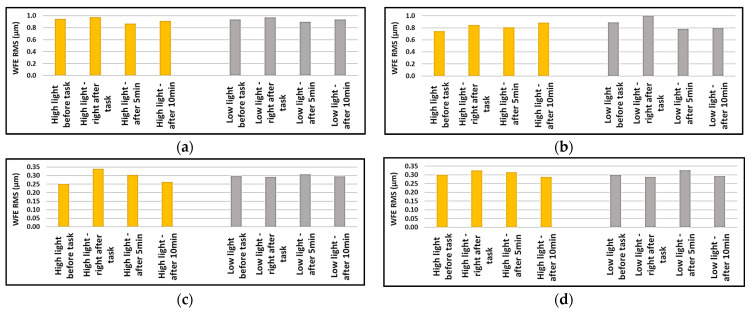
Medians of the root mean squares of aberrations for right and left eyes in high- and low-lighting conditions, before, right after, after 5 min, and after 10 min of near-vision task: (**a**) median of total RMS for right eyes; (**b**) median of total RMS for left eyes; (**c**) median of RMS of HOA for right eyes; (**d**) median of RMS of HOA for right eyes.

The root mean squares of the data were also calculated but Friedman’s test did not find statistically significant changes for any of the calculated RMS values (*p* > 0.05) before and after the reading task in different lighting conditions; however, since they were not distributed normally, the medians were plotted to compare the insignificant changes. It can be seen from Figure 6 that the total RMS of aberrations increased after finishing the reading task and then decreased in subsequent measurements. The same could be said about the RMS of HOA in high lighting condition.

**Figure 7:**
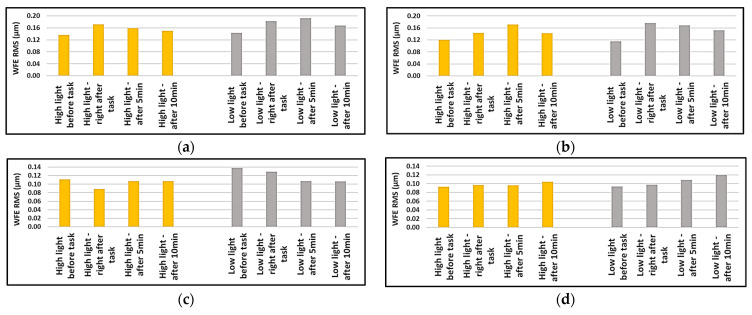
Medians of the root mean squares of higher order aberrations for right and left eyes in high- and low-lighting conditions, before, right after, after 5 min, and after 10 min of near-vision task: (**a**) median of RMS of comatic aberrations for right eyes; (**b**) median of RMS of comatic aberrations for left eyes; (**c**) median of RMS of spherical-like aberrations for right eyes; (**d**) median of RMS of spherical-like aberrations for right eyes.

From Figure 7, we can almost say that the RMS of comatic aberrations showed an increase after the reading task and recovered in 5- and 10 min after the aberrometric measurements. The RMS of spherical-like aberrations (4th and 6th order) in the high lighting condition decreased after the task and recovered in 5- and 10 min after aberrometric measurements but the change was variable in the low lighting condition.

## 4. Discussion

This study presents the impact of sustained reading on temporal changes in the wavefront error in presbyopic subjects after a sustained reading activity. Reviewing the literature, there are studies that have investigated accommodation-related changes in eyes. Ninomiya et al. [16] reported no significant changes in the root mean square values of the total higher-order aberrations with accommodation; however, Li et al. [17] found that with 5.0 mm pupils, the RMS of total HOAs changed with accommodation. Gomes and Franco [15] showed that near-vision tasks induce changes in the RMS of LOA and HOA, up to the 7th order while reading on computer task. We could confirm these findings as we also observed a slight change in the total RMS and the RMS of HOA after a sustained near-vision task.

Studies such as Ciuffreda and Lee [14] confirmed the prevalence of NITM after sustained nearwork in young subjects. The present study could also confirm this in presbyopes since with decreasing the stimulus distance to the eyes the changes in the defocus were higher. Regarding the spherical aberration, most research groups reported that the $C_{4}^{0}$ coefficient decreases with an increase in accommodation [19,22,35,36,37,38]. Glasser and Campbell [18] reported a similar result for the change in spherical aberrations with age in vitro using a lens stretcher. López-Gil and Fernández-Sánchez [19], based on theoretical and ray-tracing calculations performed on an accommodating eye model, predicted that secondary spherical aberration rises with accommodation; however, there are other researchers who reported a decrease in $C_{6}^{0}$ similar to $C_{4}^{0}$ [16,20]. We analyzed spherical-like aberration changes, which included primary and secondary spherical aberration effects together, and we could observe a decrease in the RMS of spherical-like aberrations in the high lighting condition. In contrast to spherical aberrations, comatic aberrations exhibit complicated patterns with accommodation that differ among subjects [22] and might not even be significant [20]. In the present study, the RMS of comatic aberrations, which included vertical and horizontal aberrations, was increased after a sustained reading task and recovered.

**Figure 8:**
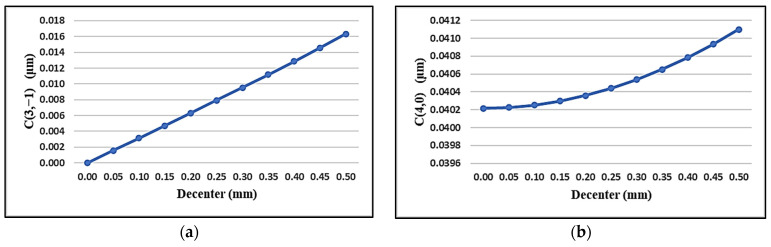
Changes in the aberrations as a function of lens decentration using Navarro’s accommodated eye model [39]: (**a**) changes in the vertical coma; (**b**) changes in spherical aberrations.

Navarro’s accommodated eye model can be used to simulate the spherical and vertical coma aberrations of the eye during accommodation as a function of the lens’s downward decentration. Figure 8 shows that for 4 diopters of accommodation (corresponding to 25 cm near stimuli) and a 4 mm pupil diameter, the change in the vertical coma and spherical aberrations follow the linear and quadratic trends with lens decentration, respectively. If we consider that changes in some of the aberration coefficients after sustained nearwork could be due to lens decentration resulting from relaxed zonules, one could predict that, for instance, for a 0.5 mm downward displacement of the crystalline lens, the $C_{3}^{-1}$ and, $C_{4}^{0}$ coefficients’ change due to 4 diopters of accommodation should be about 0.001 μm and 0.016 μm, respectively. These values are in the range of the ΔZ_1_ changes seen in Figure 5.

By simulating the aberration changes due to lens decentration—caused by relaxed zonules during 4 D accommodation—in an eye model, it was found that the expected range of changes for the vertical coma and spherical aberrations are in the order of 0.001 μm and 0.01 μm, respectively, which could justify why the observed changes were not statistically significant. These are consistent with our previous study [31] which showed slight increases in the RMS of HOA and LOA after sustained reading tasks on young subjects.

Orduna et al. [31] also investigated the effect of environmental illuminations on the ocular aberration changes during sustained reading and found that the RMS of LOA and HOA were lower in higher illumination conditions on an iPad. In the present study, the RMS of aberrations in the low lighting in some cases follows the same trends observed in the corresponding higher lighting condition and in some cases shows a different pattern of changes.

Moreover, small changes in some of the Zernike coefficients could be detected, demonstrating some predominant patterns that deserve further investigation. The potential explanation for this phenomenon is that the crystalline lens drops downward during accommodation due to gravity, which leads to changes in higher-order aberrations. Additionally, the morphological deformation during sustained accommodation might have some inertia after the reading task stops. During the recovery period after the task, it is expected that the crystalline lens reshapes back to its relaxed state with different delays in partially presbyopic subjects due to the changing biomechanical properties of the lens. Therefore, it is observed that different eyes might show different relaxation times to recover and aberrometry might be able to measure those differences.

**Figure 9:**
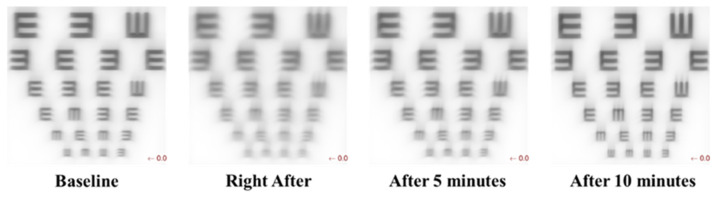
Visual quality of a pre-presbyope eye (subject JG, 47-year-old) before and after the reading task.

Increased levels of higher-order aberrations create retinal blur, and Buehren and Collins [40] found that this blurriness of retinal image quality could be perceptible. Cywinski et al. [25] also confirmed that HOAs have a small impact on the overall ocular aberrations, which are comparatively lower than LOAs but still may lead to glare and blurring of vision. The visual quality of one of the pre-presbyope subjects’ eyes can be simulated as well, using wavefront error data of the eye. It can be seen that the visual quality of the eye decreases right after finishing the near-vision task and recovers with time afterward. This indirectly confirms that relaxation of accommodation is not immediate in incipient presbyopes and the disaccommodation mechanism has hysteresis in recovering the lens shape to the normal state in these people. This justifies the blurring effects subjectively reported by subjects that are illustrated in the computational image quality example displayed in Figure 9 from wavefronts acquired in this experiment.

Our simulation of the visual quality of presbyopic eyes using the measured wavefront errors clearly demonstrated that sustained reading causes transient blurriness of vision, which recovers after a few minutes. This is contradictory to the results of Wolffsohn et al., where robustness of the insipient presbyopic accommodative system to fatigue during sustained nearwork was reported [41].

## 5. Conclusions

The present study investigated the ocular aberration changes on presbyopes after sustained reading and during recovery in different lighting conditions for the first time, to the best of the authors’ knowledge. Small changes in the Zernike coefficients could be detected after a sustained visual task under different lighting conditions, demonstrating some predominant patterns. It was also found that the total RMS of aberrations and the RMS of HOA increased immediately after finishing the near-vision reading task, and then decreased while the crystalline lens reshaped to its relaxed condition. The RMS of comatic aberrations showed a similar trend of changes but the RMS of spherical-like aberrations demonstrated an opposite trend. The effect of illumination could not be verified in this study and needs further investigation. The transient effect of HOA on the visual quality of pre-presbyope eyes were demonstrated using wavefront data. The dynamic changes in HOA reported here might be associated with the biomechanical properties and changes in decentration of the crystalline lens after sustained near-vision tasks in presbyopic subjects. This study confirms that sustained near-reading tasks are associated with measurable changes in higher-order aberrations and that might be different for tasks performed under high- and low-lighting conditions. Despite showing some predominant patterns under some conditions, the responses seem to present significant inter-subject and inter-ocular variability. In general, the variability of triplicated measures under these conditions is low but the potential impact of small misalignments while fixating the internal extended object in the aberrometer might induce additional variability.

## Figures and Tables

**Table 1:** Frequencies of the change patterns observed under the different conditions among the study population.

Zernike Coefficient	Observed Pattern	Frequency of the Observed Pattern (%)
C(2, 0)	Inverse V-shape	42 (H ^1^)–54 (L ^2^)
C(4, 0)	V-shape	46 (H)–27 (L)
C(3, −1)	V-shape	42 (H)–73 (L)

^1^ in high lighting condition. ^2^ in low lighting condition.

## Data Availability

The data presented in this study are available on request from the corresponding author.
